# A comparison of rates and severity of chronic kidney disease in deceased-donor and living-donor liver transplant recipients: times matter

**DOI:** 10.3906/sag-2007-82

**Published:** 2021-04-30

**Authors:** Yücel YANKOL, Emily BUGEAUD, Tiffany ZENS, Michael RIZZARI, Nesimi MECİT, Glen E. LEVERSON, David FOLEY, Joshua D. MEZRICH, Turan KANMAZ, Oya M. ANDAÇOĞLU, Anthony M. D’ALESSANDRO, Koray S. ACARLI, Münci KALAYOĞLU, Luis A. FERNANDEZ

**Affiliations:** 1 Department of Surgery–Division of Transplantation, School of Medicine and Public Health, University of Wisconsin, Madison, WI USA; 2 Organ Transplant Center, Memorial Şişli Hospital, İstanbul Turkey; 3 Multi-Organ Transplant Institute, Ochsner Health System, New Orleans, LA USA; 4 Transplant Institute, Henry Ford Health System, Detroit, MI USA; 5 Organ Transplant Center, School of Medicine, Koç University, İstanbul Turkey; 6 Department of Surgery–Biostatistics, School of Medicine and Public Health, University of Wisconsin, Madison, WI USA

**Keywords:** Living-donor liver transplantation, deceased-donor liver transplantation, chronic kidney disease, outcomes, mortality, graft survival

## Abstract

**Background/aim:**

The progression of chronic kidney disease (CKD) in recipients of living-donor liver transplant (LDLT) compared to deceased-donor liver transplant (DDLT) has not been studied in the literature. We hypothesize that CKD stage progression in LDLT recipients is reduced compared to that of their DDLT counterparts.

**Materials and methods:**

A retrospective study was undertaken including 999 adult, single-organ, primary liver transplant recipients (218 LDLT and 781 DDLT) at 2 centers between January 2003 and December 2012, in which CKD progression and regression were evaluated within the first 3 years after transplantation.

**Results:**

Waiting time from evaluation to transplantation was significantly lower in LDLT patients compared to recipients of DDLT. CKD stage progression from preoperative transplant evaluation to transplantation was significantly greater in DDLT. Deceased-donor liver transplant recipients continued to have higher rates of clinically significant renal disease progression (from stage I–II to stage III–V) across multiple time points over the first 3 years posttransplant. Furthermore, a greater degree of CKD regression was observed in recipients of LDLT.

**Conclusion:**

It can be concluded that LDLT provides excellent graft and patient survival, significantly reducing the overall incidence of clinically significant CKD stage progression when compared to DDLT. Moreover, there is a significantly higher incidence of CKD stage regression in LDLT compared to DDLT. These observations were maintained in both high and low model for end-stage liver disease(MELD)populations. This observation likely reflects earlier access to transplantation in LDLT as one of the contributing factors to preventing CKD progression.

## 1. Introduction 

During the past few decades, outcomes of liver transplantation (LT) have dramatically improved, but many patients still die on waiting lists (WL) due to the current shortage of suitable organs [1].One strategy used to counter balance the organ shortage has been the utilization of living-donor liver transplants (LDLT). Studies consistently demonstrate that LDLT are equivalent to deceased-donor liver transplants (DDLT) in terms of patient survival (PS) and graft survival (GS)[2–4].

With better outcomes, the transplant community turns its attention to the long‑term complications associated with transplant and immunosuppression, such as chronic medical conditions, opportunistic infections, and malignancy, in order to optimize patients’ quality of life after transplant. Both preoperative and postoperative chronic kidney disease (CKD) and end‑stage renal disease (ESRD) are common problems following LT and carry significant morbidity and mortality [5–8]. The incidence of ESRD after LT has been calculated at 2.6%, 7.5%, and 18% at 5, 10, and 20 years posttransplant. Furthermore, the overall incidence of CKD progression in this patient population has been documented to be 28% at 3 years, 40% at 5 years, and 53% at 10 years [9]. Studies have demonstrated that both preoperative (the presence and duration of pretransplant CKD, diabetes mellitus [DM], age, hyperlipidemia, hypertension, and hepatitis C) and postoperative (calcineurin inhibitor [CNI] toxicity, acute kidney injury [AKI], renal infections, prolonged ischemia, and hemodynamic instability) risk factors play a role in the progression of CKD in LT recipients[9–11]. The model for end-stage liver disease (MELD) system has led to a decrease in WL mortality by assigning priority to patients with renal dysfunction. As a result, the number of patients receiving LT with ESRD and CKD has increased [12,13]. 

Given the prevalence of CKD in the LT population and the significant morbidity and mortality associated with this disease process, we examined the relationship between CKD in LDLT recipients compared to DDLT recipients. We hypothesized that LDLT results in expedited transplantation and superior renal outcomes in terms of both CKD progression and regression when compared to DDLT. 

## 2. Materials and methods

### 2.1. Patients

Following both Institutional Review Board approvals, 999 adult primary LT recipients’ retrospective data (218 LDLT and 781 DDLT, performed between January 2003 and December 2012) were collected from one center in the United States (US) and one center in Turkey. Patients receiving simultaneous liver-kidney transplant (SLKT), those with neuroendocrine tumors, tumors beyond the Milan criteria, or stage V CKD at the time of evaluation were excluded. In addition, patients who had liver failure and stage V CKD with hepato-renal syndrome for which SLKT was indicated were excluded from posttransplant analysis. Both country have similar organ allocation systems. Three percent of LDLT (n = 6) and 89% of DDLT (n = 695) included in the study were performed in the US. In contrast, 97% of LDLT (n = 212) and 11% of DDLT (n = 86) in this series were transplanted in Turkey. 

### 2.2. Kidney function and stage progression/regression

Chronic kidney disease is defined as reduced glomerular filtration rate (GFR) for 3 months or more with GFR determined by the modification of diet in renal disease equation. The National Kidney Foundation (NKF) defines the stages of CKD as follows: stage I: GFR > 90 mL/min per 1.37m2; stage II: GFR 60–89 mL/min per 1.37m2; stage III: GFR 30–59 mL/min per 1.37m2; stage IV: GFR 15–29 mL/min per 1.37m2; and stage V: GFR <15 mL/min per 1.37m2 or dialysis-dependent [5]. CKD progression past stage III was determined to be clinically significant. This clinical cutoff was established based on the current recommendations of the American Society of Nephrology (ASN), which state that patients with CKD beyond stage III should be referred to a nephrologist for formal evaluation.

The data for preoperative CKD stage were based on an average of 3 outpatient serum creatinine (SCr) values for each patient obtained at the time of initial evaluation for transplantation and immediately prior to transplantation. Postoperative CKD was evaluated at 3, 6, 9, 12, 24, and 36 months posttransplantation. Patients were included in the analysis if they had a postoperative result at 1 month ± 1 week, 3 months ± 2 weeks, 6 months ± 1 month, 12 months ± 1 month, and 36 months ± 3 months. If the patient had no results within these time points, he or she was excluded from the analysis (represented as missing data points), but the patient was still followed for the remainder of the study. Using these SCr values, we calculated patient GFRs and assigned CKD stages using the NKF definitions. Patients who died after the first year posttransplant and did not have CKD progression prior to death were censored at the time of death. If they had disease progression prior to death, they were considered to have an event at the time of death.

### 2.3. Immunosuppression

The protocols for immunosuppressive therapy were identical at both institutions. Similar triple maintenance immunosuppressive therapy consisted of prednisone, CNI (Tacrolimus, Prograf, Astellas Pharma US Inc., Deerfield, IL, USA), and mycophenolate mofetil (CellCept, Roche Laboratories, Nutley, NJ, USA). Tacrolimus levels were analyzed at 1, 3, 6, 12, and 36 months postoperatively. 

### 2.4.Outcomes

The primary outcomes were CKD progression and regression. These were evaluated as both progression/regression from one stage to the other and progression/regression to clinically significant renal dysfunction. When a patient progressed/regressed between stage I–II and stage III–V, they were considered to have a clinically significant progression/regression of their CKD based on the morbidity and mortality factors and recommendations of ASN. 

In order to evaluate the CKD progression/regression rate between DDLT and LDLT, this analysis was stratified based on different periods: from evaluation to transplantation and from evaluation to the first and third years posttransplant. In order to compare both recipient groups, the data were analyzed based not only on the cumulative incidence between the groups regardless of CKD stage, but also according to patients’ CKD stage, allowing an individual stage-by-stage comparison between both groups. All cases were stratified into 2 groups according to MELD score at the time of transplantation. All of the MELD scores used for analysis were physiological MELD scores calculated using recipient creatinin, total bilirubin, and international normalized ratio (INR) values at the time of transplantation. Exception MELD scores for special diseases were not used in the analysis. The cutoff was determined based on previous publications in which a mean MELD score lower than 25 was associated with a 90-day mortality on the waiting list (WL) of less than 16%, and a higher MELD score was associated with an unacceptably high mortality risk (>25% at 30 days) [14]. Finally, analysis of CKD progression/regression was also performed by dividing the recipients according to physiological MELD score (MELD < 25 and MELD ≥ 25) at the time of transplant. Secondary outcomes analyzed in this study also included PS and GS between the 2 recipient groups. 

### 2.5. Statistics

In comparing patient characteristics between the live-donor and deceased-donor groups, t-tests were used for continuous variables and Fisher’s exact test or chi-square tests were used for categorical variables. Stage progression/regression of CKD was compared between groups using Fisher’s exact test. Univariate and multivariate models to assess potential risk factors of CKD progression were evaluated using logistic regression. Tacrolimus levels were compared between groups using t-tests. Graft survival and patient survival rates were estimated utilizing the Kaplan–Meier method and compared between groups with log-rank tests. All analyses were performed using SAS statistical software (SAS Institute, Inc., Cary, NC, USA) and P values less than 0.05 were considered to be statistically significant.

## 3. Results

### 3.1. Demographics

Deceased donors were older (P = 0.001) and had higher BMIs (P = 0.001) than live donors. Other donor characteristics were not statistically different. Deceased-donor recipients were also older, had higher BMIs (P = 0.001), statistically significant increased WL time (400 days vs. 65, P = 0.001), and higher MELD scores (P = 0.001). The etiology of liver disease in DDLT recipients was more likely to be HCV or alcoholic cirrhosis, while LDLT recipients were HBV. Finally, DDLT recipients demonstrated significantly higher CKD stages at evaluation and transplantation (Table 1). 

**Table 1:** Comparison of donor and recipient demographics with tacrolimus levels comparison of recipients after DDLT and LDLT.

	DDLT (n=781)		LDLT (n=218)	p-value
Donor Demographics				
Mean Age (years)	42± 16.7		34.4 ± 10.7	0.001
Sex (n)				
Female	284 (36.4%)		93 (42.7%)	0.13
Male	497 (63.6%)		125 (57.3%)	
BMI	27.5± 6.5		25.6 ± 3.7	0.001
Graft (n)				
DBD	711(91%)		-	-
DCD	70 (9%)		-	
Recipient Demographics and Postoperative Tacrolimus Levels				
Mean Age (years)		54 ± 10	50 ± 12	0.001
Sex				
Female		270 (34.6%)	81 (37.2%)	0.5
Male		511 (65.4%)	137 (62.8%)	
BMI		28.8 ± 6.1	26.1± 4.5	0.001
MELD (n)				
Mean MELD		23 ± 8	16 ± 7	0.001
<25		512 (65.6%)	197 (90.4%)	
≥ 25		269 (34.4%)	21(9.6%)	
Mean Waitlist Time (days)		400 ± 672	65 ± 126	0.001
Etiology of Liver Failure (n)				
HCV		218 (27.6%)	36 (16.5%)	0.001
Alcoholic cirrhosis		346 (43.8%)	23 (10.6%)	0.001
HBV		35 (4.4%)	81(37.2%)	0.001
Cryptogenic		43 (5.4%)	19 (8.7%)	0.08
Primary sclerosing cholangitis		52 (6.6%)	11(5.1%)	0.53
Primary biliary cirrhosis		32 (4.1%)	7 (3.2%)	0.69
Primary malignancy		173 (21.9%)	39 (17.9%)	0.22
Other		148 (18.7%)	43 (19.7%)	0.77
CKD stage at evaluation (n)				
Stage I		169 (21.7%)	140 (64%)	0.001
Stage II		325 (41.6%)	54 (24.9%)	
Stage III		192 (24.6%)	18 (8.3%)	
Stage IV		68 (8.7%)	5 (2.3%)	
Stage V		27 (3.4%)	1 (0.5%)	
Missing data point		0	0	
CKD stage at transplantation (n)				
Stage I		152 (19.4%)	147 (67.4%)	0.001
Stage II		251(32.3%)	45 (20.6%)	
Stage III		244 (31.2%)	19 (8.7%)	
Stage IV		94 (12%)	6 (2.8%)	
Stage V		40 (5.1%)	1 (0.5%)	
Missing data poin		0	0	
Tacrolimus Level				
1 month post-transplant		8.4 ± 5.1	12.1 ± 5.1	0.001
3 months post-transplant		8.2 ± 4.5	11.2 ± 4.2	0.001
6 months post-transplant		7.0 ± 4.0	9.3 ± 3.6	0.001
1 year post-transplant		6.4 ± 3.5	8.2 ± 3.1	0.001
3 years post-transplant		5.4 ± 2.8	6.3± 2.2	0.012

*DDLT: deceased-donor liver transplant, LDLT: living-donor liver transplant, CKD: chronic kidney disease, BMI: body mass index, DBD: donor after brain death, DCD: donor after cardiac death, MELD: model for end-stage liver disease, HCV: hepatitis C virus, HBV: hepatitis B virus. #Variables: mean ± SD and n (%).

### 3.2. CKD progression/regression

Progression/regression of CKD were examined from the time of evaluation to transplantation. DDLT recipients with lower stages of KD at evaluation (stages I–III) had increased CKD progression compared to their LDLT counterparts. LDLT recipients were found to more frequently regress by one or more stages of CKD if their initial CKD was stage II or III. In the LDLT group, there were not enough stages IV and V patients to make an analysis and to compare with the DDLT group. We could not determine P values for both progression and regression in the comparison of stages IV and V. Furthermore, DDLT recipients (31.8%) had a greater rate of clinically significant progression of CKD (stage II–III to stage III–V) compared to LDLT recipients (8.3%) (P = 0.001) (Table 2). In addition, a total of 123 patients needed dialysis at the time of transplantation. Among those, 115 were DDLT and 8 were LDLT recipients. Furthermore, among the 754 patients in the DDLT group that were at CKD stages I to IV, 36 received SLKT (4.8%). Among the LDLT, 218 patients had a CKD between stages I and IV, and no patients required SLKT.

**Table 2:** CKD progression and regression from transplant evaluation to transplantation.

CKD Progression and regression: From transplant evaluation to transplantation					
	n	DDLT Outcome	n	LDLT Outcome	p-value
Patients demonstrating progression of CKD by at least one stage (n):					
Stage I	169	78 (46.2%)	140	18 (12.9%)	0.001
Stage II	325	125 (38.5 %)	54	8 (14.8%)	0.001
Stage III	192	39 (19.2%)	18	0 (0%)	0.046
Stage IV	68	14 (20.6 %)	5	0 (0%)	-
Stage V	27		1		-
Missing data point	0		0		
Patients demonstrating regression of CKD by at least one stage (n):					
Stage V	27	12 (44.4%)	1	1 (100%)	-
Stage IV	68	23 (33.8%)	5	3 (60%)	-
Stage III	192	39 (20.3%)	18	11 (61.1%)	0.001
Stage II	325	42 (12.9%)	54	16 (29.6%)	0.004
Stage I	169		140		
Missing data point	0		0		
Patients demonstrating clinically significantprogression of CKD (n):[Stage (I- II) to Stage (III-V)]	494	157 (31.8%)	194	16 (8.3%)	0.001

Progression/regression of CKD was also evaluated from the time of evaluation to 1 year postoperatively. During this period, DDLT recipients were more likely to have clinically significant CKD progression (42.8%) compared to LDLT recipients (26.8%) (P = 0.001); LDLT recipients were more likely to have clinically significant CKD regression (70.6%) compared to DDLT recipients (28.2%) (P = 0.001). Additionally, patients in the LDLT group with stage III CKD were more likely to have regression (69.2%) compared to DDLT recipients with stage III CKD (30.9%) (P = 0.01). There were not enough stages IV and V patients in the LDLT group to make an analysis and to compare with the DDLT group. We could not have P values for both progression and regression in the comparison of stages IV and V. Other comparisons in regard to rate of CKD progression or regression according to specific CKD stages were not statistically significantly different (Table 3).

**Table 3:** CKD progression and regression from evaluation to 1–3 year posttransplant.

CKD progression and regression: From transplant evaluation to 1 year post-transplant					
	n	DDLT Outcomes	n	LDLT Outcomes	p-value
Patients demonstrating progression of CKD by at least one stage (n):					
Stage I	129	101 (78.3%)	115	83 (72.2%)	0.3
Stage II	264	128 (48.5%)	42	15 (35.7 %)	0.14
Stage III	152	14 (9.2%)	13	0 (0%)	0.6
Stage IV	54	0 (0%)	4	1 (25%)	-
Stage V	21		0		
Missing data point	161		41		
Patients demonstrating regression of CKD by at least one stage (n):					
Stage V	21	21 (100%)	0	0 (0%)	-
Stage IV	57	51 (89.5%)	4	3 (75%)	-
Stage III	152	47 (30.9%)	13	9 (69.2%)	0.01
Stage II	267	19 (7.1%)	42	6 (14.3%)	0.13
Stage I	129		115		
Missing data point	155		44		
Patients demonstrating clinically significant progression of CKD (n):[Stage (I- II) to Stage (III-V)]	393	168 (42.8%)	157	42 (26.8%)	0.001
Patients demonstrated clinically significantregression of CKD (n):[Stage (III-V) to Stage (I-II)]	227	64 (28.2%)	17	12 (70.6%)	0.001
CKD progression and regression: From transplant evaluation to 3 years post-transplant					
Patients demonstrating progression of CKD by at least one stage (n):					
Stage I	101	81 (80.2%)	44	35 (79.6%)	1.0
Stage II	209	115 (55.2 %)	12	6 (50 %)	0.77
Stage III	112	10 (8.9%)	2	0 (0%)	-
Stage IV	44	1 (2.3%)	1	0 (0%)	-
Stage V	16		0		
Missing data point	299		159		
Patients demonstrating regression of CKD by at least one stage (n):					
Stage V	16	16 (100%)	0	0 (0%)	-
Stage IV	47	41 (87.2%)	1	1 (100%)	-
Stage III	112	30 (26.8%)	2	1 (50%)	-
Stage II	221	6 (2.8%)	12	2 (16.7%)	0.06
Stage I	101		44		
Missing data point	284		159		
Patients demonstrating clinically significant progression of CKD (n):[Stage (I- II) to Stage (III-V)]	310	146 (47.1%)	56	12 (21.4%)	0.001
Patients demonstrating clinically significantregression of CKD (n):[Stage (III-V) to Stage (I-II)]	172	45 (26.2%)	3	2 (66.7%)	-

Progression/regression of CKD was also analyzed from the time of evaluation to 3 years postoperatively. At the 3-year time point, the only significant difference between the 2 groups was that DDLT recipients were more likely to have clinically significant progression of CKD (47.1%) compared to LDLT recipients (21.4%) (P = 0.001). Other comparisons in regard to overall incidence or rates of CKD progression or regression according to specific CKD stages were not statistically significantly different at 3 years. There were not enough stages III,IV, and V patients in the LDLT group to compare with the DDLT group. We could not have P valuesfor the comparison for both progression/regression of at least 1stage in stages III, IV, V, or clinically significant regression of CKD (Table 3).

In addition, progression/regression of CKD was analyzed from transplantation to 1 year postoperatively. DDLT recipients were more likely to have clinically significant progression of CKD (32.9%) compared to LDLT recipients (23.2%) (P = 0.03). There were not enough stage IV and V patients in the LDLT group to make an analysis and to compare with the DDLT group. We could not have P values for both progression and regression forthe comparison of stages IV and V (Table 4). 

**Table 4:** CKD progression and regression from transplant to 1–3 years posttransplant.

CKD progression and regression: From transplantation to 1 year post-transplant					
	n	DDLT Outcomes	n	LDLT Outcomes	p-value
Patients demonstrating progression of CKD by at least one stage (n):					
Stage I	115	83 (72.2%)	121	85 (70.3%)	0.77
Stage II	204	78 (38.2 %)	34	12 (35.3 %)	0.85
Stage III	199	12 (6%)	15	0 (0%)	1.0
Stage IV	71	4 (5.6%)	3	3 (100%)	-
Stage V	31		1		
Missing data point	161		1		
Patients demonstrating regression of CKD by at least one stage (n):					
Stage V	55	52 (94.6%)	1	1 (100%)	-
Stage IV	83	74 (89.2%)	3	0 (0%)	-
Stage III	204	57 (27.9%)	15	7 (46.7%)	0.14
Stage II	205	15 (7.3%)	34	4 11.8(%)	0.32
Stage I	117		121		
Missing data point	117		41		
Patients demonstrating clinically significant progression of CKD (n):[Stage (I- II) to Stage (III-V)]	319	105 (32.9%)	155	36 (23.2%)	0.03
Patients demonstrating clinically significantregression of CKD (n):[Stage (III-V) to Stage (I-II)]	216	85 (28.2%)	19	8 (42.1%)	0.20
CKD progression and regression: From transplantation to 3 years post-transplant					
Patients demonstrating progression of CKD by at least one stage (n)					
Stage I	82	65 (79.3%)	41	32 (78.1%)	1.0
Stage II	162	72 (44.4 %)	14	4 (28.6 %)	1.0
Stage III	152	9 (5.9%)	4	0 (0%)	-
Stage IV	48	1 (2.1%)	0	0 (0%)	-
Stage V	22		0		
Missing data point	315		159		
Patients demonstrating regression of CKD by at least one stage (n):					
Stage V	36	34 (94.4%)	0	0 (0%)	-
Stage IV	56	49 (87.5%)	0	0 (0%)	-
Stage III	156	36 (23.1%)	4	2 (50%)	-
Stage II	163	12 (7.4%)	14	2 (14.3%)	0.31
Stage I	84		41		
Missing data point	286		159		
Patients demonstrating clinically significant progression of CKD (n):[Stage (I- II) to Stage (III-V)]	244	98 (40.2%)	55	11 (20%)	0.005
Patients demonstrating clinically significantregression of CKD (n):[Stage (III-V) to Stage (I-II)]	222	59 (26.6%)	4	2 (50%)	

From transplantation to 1 year posttransplant: DDLT recipients were more likely to have clinically significant progression of CKD (32.9%) compared to LDLT recipients (23.2%). From transplantation to 3 years posttransplant: Stage of CKD represents the stage at transplant. DDLT recipients were more likely to have clinically significant progression of CKD (40.2%) compared to LDLT recipients (20%).

Finally, CKD progression/regression was evaluated from transplantation to 3 years postoperatively. DDLT recipients were more likely to have clinically significant progression of CKD (40.2%) compared to LDLT recipients (20%) (P = 0.005). There were not enough stages III, IV, and V patients in the LDLT group to compare with the DDLT group. We could not have P values for the comparison of both progression and regression for at least 1 stage in stages III, IV, V, or clinically significant regression of CKD (Table 4).

### 3.3. MELD

Groups were compared controlling for preoperative MELD score.For patients with MELD scores <25, DDLT recipients had a statistically significantly greater rate of CKD progression than LDLT recipients at the following points: a) evaluation to transplantation (22.6% vs. 7.5%, P = 0.001); b) evaluation to 1 year posttransplant (40.1% vs. 27.7%, P = 0.014); and c) evaluation to 3 years posttransplant (46.2% vs. 23.4%, P = 0.004), respectively. A larger percentage of LDLT recipients saw clinically significant regression of CKD stage from evaluation to transplantation (63.6% vs. 20.1%, P = 0.001), as well as from evaluation to 1 year posttransplant in the MELD <25 group (68.8% vs. 33.6%, P = 0.012). Similarly, for patients with initial MELD ≥25, more patients demonstrated clinically significant progression of CKD at the following time points: a) evaluation to transplantation (58% vs. 15.8%, P = 0.008); b) evaluation to 1 year posttransplant (50.5% vs. 18.8%, P = 0.028); and c) evaluation to 3 years posttransplant (46.2% vs. 23.4%, P =0.034), respectively. In addition, 100% of patients in the LDLT group showed clinically significant regression; however, the numbers are too small to draw clinical conclusions (Table 5).

**Table 5:** Clinically CKD progression and regression according to low and high MELD score.

Clinically significant CKD progression and regression according to low and high MELD scores					
	n	DDLT Outcomes	n	LDLT Outcomes	p-value
MELD<25					
Patients demonstrating clinically significant [Stage (I- II) to Stage (III-V)] progression of CKD					
Evaluation to transplantation (n)	341	77 (22.6%)	161	13 (7.5%)	0.001
Evaluation to 1 yr post-tx (n)	294	118 (40.1%)	141	39 (27.7%)	0.014
Evaluation to 3 yrs post-tx (n)	238	110 (46.2%)	47	11 (23.4%)	0.004
Transplantation to 1 yr post-tx (n)	262	81 (30.9%)	141	33 (23.4%)	0.13
Transplantation to 3 yrs post-tx (n)	207	80 (30.9%)	47	10 (21.3%)	0.03
Patients demonstrating clinically significant [Stage (III-V) to Stage (I-II)] regression of CKD					
Evaluation to transplantation (n)	146	30 (20.1%)	22	14 (63.6%)	0.001
Evaluation to 1 yr post-tx (n)	122	41 (33.6%)	16	11 (68.8%)	0.012
Evaluation to 3 yrs post-tx (n)	96	23 (24%)	2	1 (50%)	-
Transplantation to 1 yr post-tx(n)	172	45 (26.2%)	16	5 (31.3%)	0.77
Transplantation to 3 yrs post-tx (n)	134	28 (20.9%)	2	5 (31.3%)	-
MELD≥25					
Patients demonstrating clinically significant [Stage (I- II) to Stage (III-V)] progression of CKD					
Evaluation to transplantation (n)	124	72 (58%)	19	3 (15.8%)	0.008
Evaluation to 1 yr post-tx (n)	99	50 (50.5%)	16	3 (18.8%)	0.028
Evaluation to 3 yrs post-tx (n)	72	36 (50%)	9	1 (11.1%)	0.034
Transplantation to 1 yr post-tx (n)	58	24 (41.1%)	14	3 (21.4%)	0.23
Transplantation to 3 yrs post-tx (n)	38	19 (50%)	8	1 (12.5%)	0.11
Patients demonstrating clinically significant [Stage (III-V) to Stage (I-II)] regression of CKD					
Evaluation to transplantation (n)	124	18 (14.5%)	2	0 (0%)	
Evaluation to 1 yr post-tx (n)	105	23 (21.9%)	1	1(100%)	-
Evaluation to 3 yrs post-tx (n)	54	22 (29%)	1	1(100%)	-
Transplantation to 1 yr post-tx (n)	165	48 (29.1%)	3	3 (100%)	-
Transplantation to 3 yrs post-tx (n)	110	39 (35.5%)	2	2 (100%)	-

### 3.4. Univariate and multivariate analyses

Univariate and multivariate analyses were performed to determine potential independent variables affecting CKD progression/regression evaluation to transplantation. Pretransplant DM was found to have the most significant effect, with an odds ratio (OR) of 4.15 (95% CI 2.43–7.08, P = 0.001) in the univariate and 2.5 (95% CI 0.76–2.54, P = 0.003) in the multivariate analysis. Physiological MELD at the time of transplant was found to have an OR of 0.94 (95% CI 0.92–0.96, P = 0.001) in the univariate and 0.95 (95% CI 0.92–0.99, P = 0.007) in the multivariate analysis. Waiting time prior to transplantation was also a significant factor contributing to CKD progression, with an OR of 0.99 (95% CI 0.99–1.0, P = 0.001) in the univariate and a similar OR of 0.99 (CI 0.99–1, P = 0.028) in the multivariate analysis. The OR listed with waiting list time is very close to 1, since it represents the change in the odds of CKD associated with a 1-unit increase in waiting time (Table 6).

**Table 6:** Univariate and multivariate analysis: evaluation of all factors which are independent predictors of progression of CKD from evaluation to transplantation.

Univariate and multivariate analysis of factors which are predictors of progression of CKD from evaluation to transplantation			
	Odds Ratio	Confidence Interval	p-value
Univariate Analysis			
Pretransplant DM	4.15	2.43 to 7.08	0.001
Live donor	3.40	2.17 to 5.32	0.001
HCV	0.82	0.58 to 1.15	0.24
White	1.34	0.76 to 2.35	0.32
Age >55	0.74	0.55 to 0.99	0.048
MELD	0.94	0.92 to 0.96	0.001
Waitlist time	0.99	0.99 to 1.00	0.001
Multivariate Analysis			
Pretransplant DM	2.5	0.76 to 2.54	0.003
Live donor	1.39	0.76 to 2.54	0.28
HCV	1.38	0.75 to 2.53	0.30
White	1.04	0.37 to 2.93	0.94
Age >55	0.82	0.50 to 1.36	0.45
MELD	0.95	0.92 to 0.99	0.007
Waitlist time	0.99	0.99 to 1.00	0.028

### 3.5. Tacrolimus

Given its known nephrotoxic effect, tacrolimus levels were compared in both groups at 1, 3, 6, 12, and 36 months posttransplant. This comparison was performed to eliminate the potential argument that LDLT recipients were managed with lower levels of CNI, in which case the findings described above could possibly be explained as a bias introduced in their postoperative immunosuppressive management. However, LDLT recipients were managed with significantly higher serum levels of CNI at all time points (Table 1).

### 3.6. Patient and graft survival

No statistically significant differences were observed between recipients of DDLT and LDLT (P = 0.14 and P = 0.77, respectively) (Figure). 

**Figure:**
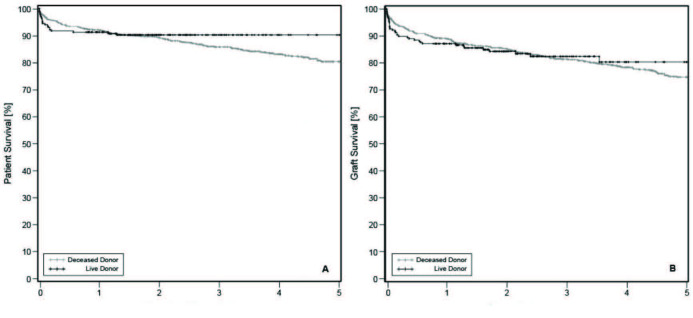
Patient (A) and graft (B) survival comparison with Kaplan–Meier.

### 3.7. Living-donor complications

The overall complication rate for LD was 19.3% (42/218). According to the Dindo/Clavien score, 50% of complications were Grade 1 (n = 21), mostly wound infections and ascites which resolved with medical management. Eight patients developed Grade 2 complications (3 cases of postoperative ileus requiring NG tube and IV fluid resuscitation, 2 biliary leaks at the cut surface that spontaneously resolved, and 2 minor pulmonary embolisms requiring prolonged anticoagulation). A total of 13 patients developed Grade 3 complications requiring surgical intervention without sequelae (4 percutaneous drainage for pleural effusion and abdominal collection, 2 ERCP for biliary leak and stricture, 3 surgery for postoperative bleeding, 3 surgery for incisional hernia, 1 hepaticojejunostomy for biliary stricture). No Grade 4 complications ordeath occurred among our 218 donors.

## 4. Discussion

Advances in PS and GS over the last decade have led the transplant community to focus on understanding and controlling long-term complications associated with LT, such as CKD. The development of CKD after LT has been associated with a 4.55×increased risk of death [5,10,13]. Additionally, PS for those who undergo SLKT has been found to be statistically greater (71.4%) compared to that in patients receiving a liver only and remain dialysis-dependent (27%) [3,15]. Multiple factors contribute to CKD risk in this patient population and have a direct impact on how patients are treated in the pre-, peri-, and posttransplant settings. These risk factors include but are not limited to: a) level of pretransplant renal function; b) recipient demographics and comorbidities; c) AKI during the perioperative period; and d) long‑term CNI exposure [7,13]. 

It is known that LDLT reduces the risk of health deterioration and death on the WL[16]; however, notmany previous studies have compared the rate and severity of CKD progression/regression between DDLT and LDLT. This study demonstrates that recipient waiting time from evaluation to transplant was significantly lower in LDLT patients. Similarly, the average MELD score at transplant was significantly lower in the LDLT patients. CKD stage progression from evaluation to transplant was significantly greater in DDLT patients (31.8%) compared to LDLT recipients (8.3%). In addition, DDLT recipients continued to have higher rates of clinically significant CKDprogression (from stage I–II to stage III–V) (32.9%) than LDLT recipients (23.2%) (P = 0.03) for the first year and in DDLT recipients (40.2%), compared to 20% in the LDLT group (P = 0.005) for the third year after transplant. The rate of clinically significant progression in DDLT recipients within the first year after transplant is not a new finding. Recently, Mangus et al. observed a decrease in GFR >20 mL/min per 1.73 m2 in 42% of recipients within the first year after transplant [17].Although the rate of progression described by them is slightly higher than that described in this study, the discrepancies in rate of CKD progression between the transplant groups may likely be related to the higher number of patients in the early CKD stage in the study by the Indiana group compared to this study cohort [17]. Kang et al. reported that renal function significantly decreased the first year after LT, and that baseline renal function was an independent risk factor for worsening renal function in LT recipients. In their study, there were no correlations between renal function changes and tacrolimus serum levels [11]. Sandal et al. reported similar 10-year ESRD incidence in both DDLT and LDLT, but LDLT recipients seem to have a more sustained decline in GFR [6]. Regardless of the differences between centers, these data highlight the importance of identifying strategies to protect renal function after LT, including reinforcing the utilization of LDLT and early LT after evaluation to reduce the incidence of CKD progression. 

Furthermore, clinically significant CKD regression for DDLT recipients is lower (28.2%) at 1 year posttransplant compared to that of LDLT recipients (42.1%). These findings are in contrast to the recently-published results by Mangus et al. in which only 22% of recipients had an absolute improvement in GFR >5 mL/min per 1.73 m2 [17]. Based on these findings, it can be concluded that the decreased waiting time associated with LDLT offers significant protection against CKD progression, although analysis of a larger patient population is required to categorically confirm the study findings. 

From evaluation to transplantation, DDLT recipients with the initial diagnosis of stages I, II, or III CKD were found to have more advanced progression of CKD than LDLT recipients in the same CKD stage. During the postoperative period, however, these stage‑specific differences were no longer seen. Overall, DDLT recipients continued to have higher rates of clinically significant deterioration of renal function (from stage I–II to stage III–V). These observations suggest that it is WL time and the damage in the renal reserve that occurs during that period and not the quality of the organ itself that accounts for the worse outcomes in DDLT. Since these recipients experience significant CKD progression while awaiting transplantation, a larger proportion of patients have already progressed to clinically significant (stage III or greater) disease by the time of transplantation (48.3%) compared to their LDLT counterparts (12%). It is also important to note that 5.1% of DDLT recipients had already progressed to stage V CKD by the time of transplantation, compared to only 0.5% in the LDLT group, adding significant complexity to the transplant procedure, since the majority of these patients require SLKT. Furthermore, 32.3% of DDLT patients were reported to have stage II disease at the time of transplantation, compared to 20.6% in LDLT. In these patients, any posttransplant CKD progression will lead to clinically significant renal dysfunction. 

Patients with higher functional reserves in stage I–II CKD are known to be more likely to progress than patients with lower functional reserves in stage IV–V [11,17]. Though DDLT patients had overall worse outcomes and increased CKD progression, when LDLT and DDLT were examined separately over all time points, both patient populations demonstrated a higher percentage of CKD progression in recipients with stage I–II CKD. In addition, a higher percentage of regression was documented in patients who were in stage IV–V CKD. The current literature also suggests that MELD is protective, due to the fact that patients with higher MELD scores have lower functional reserves and are less likely to progress [9]. The data in this study revealed that DDLT recipients with higher MELD scores were more likely to have clinical progression of their disease than comparable DDLT recipients with lower MELD scores across all time points. In contrast, LDLT recipients with higher MELD scores were less likely to experience CKD progression than those with lower MELD scores during all time points, with the exception of evaluation to transplant. Again, it appears that the shorter waiting time associated with LDLT offers patients a significant benefit. Finally, the rate of regression was higher at 1 and 3 years posttransplant in both LDLT and DDLT recipients with higher MELD scores, likely because their disease was more severe at evaluation.

In order to understand the factors that contribute to CKD progression from evaluation to transplantation, univariate and multivariate analyses were performed, including all of the factors that have previously been implicated to contribute to kidney disease progression after transplantation [7,9,12,13,18]. Pretransplant diabetes, MELD score at the time of transplant, and time on the WL are all strong contributing factors that independently correlate with CKD progression. Given the increased hazard of posttransplant mortality relative to ESRD, the overriding goal in managing these patients on the WL should be to optimize strategies to allow quick access to transplantation in order to minimize morbidity and mortality risk. With the exception of pretransplant diabetes, LDLT has the potential to significantly modify those factors, since patients could be transplanted earlier and likely with lower MELD scores.

There are limitations to this study. First, the retrospective nature and missing data points in the analysis. One of the most common reasons for a missing data point was a patient who did not have height and weight documented; therefore, there was no way to calculate GFR for that time point. Second, the patient populations were obtained from hospital databases in 2 different national healthcare systems, with 89% of DDLT in the series transplanted in the US and 97% of LDLT transplanted in Turkey. Both databases, however, were built along similar concepts in order to collect similar data, and the immunosuppressive regimens were similar. Despite the fact that the LDLT patients had high average tacrolimus levels, the LDLT recipients were found to have better outcomes related to CKD progression/regression. A third limitation is that eGFR may overestimate actual GFR in LT candidates due to decreased muscle mass [19,20]. It is clear that serum creatinine SCr‑based estimates of renal function are not accurate in patients with cirrhosis, and a rise in SCr is often a late indicator of kidney injury. Patients with cirrhosis are known to have low SCr levels related to lower muscle mass, decreased production of creatinine by the liver, and potentially increased tubular secretion of creatinine related to medications commonly prescribed pretransplant [21,22]. In clinical practice, SCr testing is widely available and relatively inexpensive. Despite the lack of a consistent correlation between SCr and the GFR in the setting of cirrhosis, it is a key factor in calculating MELD score. A fourth limitation is that this data set does not include proteinuria, urine sodium concentration, or inflammatory biomarkers such as neutrophil gelatinase-associated lipoprotein (NGAL), IL-18, or others; these values might be useful predictors of renal prognosis/progression.

## 5. Conclusion

LDLT provides excellent GS and PS, significantly reducing the overall incidence of clinically significant CKD stage progression when compared to DDLT. Moreover, there is a significantly higher incidence of CKD stage regression in LDLT. These observations were maintained in both high‑ and low‑MELD populations. This observation likely reflects earlier access to transplantation in LDLT recipients rather than organ quality at transplantation between LDLT and DDLT. 

## Informed consent

Ethics committee approval for this study was received from the Institutional Review Board of the University of Wisconsin, Madison, WI, USA (No: 2012-0556- CR006, Last Renew Date: 03.12.2018). In addition, it was approved by Memorial Şişli Hospital, İstanbul, Turkey Ethical Committee (No: 11, Date: 10.08.2012). Written informed consent was obtained from all patients who participated in this study.
